# An Additional Certification as a Centre for Geriatric Trauma Had No Benefit on Mortality Among Seriously Injured Elderly Patients—An Analysis of the TraumaRegister DGU^®^ with Data of the Registry for Geriatric Trauma (ATR-DGU)

**DOI:** 10.3390/jcm13226914

**Published:** 2024-11-17

**Authors:** Bastian Pass, Rene Aigner, Rolf Lefering, Sven Lendemans, Bjoern Hussmann, Teresa Maek, Dan Bieler, Christopher Bliemel, Carl Neuerburg, Carsten Schoeneberg, the TraumaRegister DGU

**Affiliations:** 1 Department of Orthopedic and Emergency Surgery, Alfried Krupp Hospital, 45276 Essen, Germany; bastian.pass@krupp-krankenhaus.de (B.P.); sven.lendemans@krupp-krankenhaus.de (S.L.); teresa.maek@krupp-krankenhaus.de (T.M.); 2 Centre for Orthopedics and Trauma Surgery, University Hospital Giessen and Marburg, 35043 Marburg, Germany; aignerr@med.uni-marburg.de (R.A.); bliemel@med.uni-marburg.de (C.B.); 3 Institute for Research in Operative Medicine (IFOM), Witten/Herdecke University, 58455 Witten, Germany; rolf.lefering@uni-wh.de; 4 Department of Trauma Surgery, Klinikum Hochsauerland, 59759 Arnsberg, Germany; b.hussmann@klinikum-hochsauerland.de; 5 Department of Orthopaedics and Trauma Surgery, Heinrich Heine University Hospital Düsseldorf, 40225 Düsseldorf, Germany; danbieler@uni-duesseldorf.de; 6 Department for Trauma Surgery and Orthopaedics, Reconstructive and Hand Surgery, Burn Medicine, German Armed Forces Central Hospital, 56072 Koblenz, Germany; 7 Department of Orthopaedics and Trauma Surgery, Musculoskeletal University Centre Munich (MUM), University Hospital (LMU), 80336 Munich, Germany; carl.neuerburg@med.uni-muenchen.de; 8 Committee on Emergency Medicine, Intensive Care and Trauma Management (Sektion NIS) of the German Trauma Society (DGU), 51109 Cologne, Germany

**Keywords:** elderly polytrauma, orthogeriatric care, centre for geriatric trauma

## Abstract

**Background/Objectives**: The number of seriously injured elderly patients is continuously rising. Several studies have underlined the benefit of orthogeriatric co-management in treating older patients with a proximal femur fracture. The basis of this orthogeriatric co-management is a certification as a Centre for Geriatric Trauma (ATZ). Data of seriously injured patients are collected in the TraumaRegister DGU^®^ (TR-DGU) from participating trauma centres. We hypothesise that if a certified trauma centre is also a certified Centre for Geriatric Trauma, a benefit can be measured. **Methods**: Retrospective cohort analysis was conducted from 1 January 2016 to 31 December 2021. The TraumaRegister DGU^®^ collected the data prospectively. This retrospective multicentre registry study included patients 70 years or older with an abbreviated injury scale of ≥3 and intensive care unit treatment from 700 certified Trauma Centres and 110 Centres for Geriatric Trauma in Germany, Austria and Switzerland. The primary outcome was mortality in in-hospital stays. Other outcome parameters were days of intubation, the length of stay in ICU, and in-hospital stays. Furthermore, the discharge target and the Glasgow Outcome Scale (GOS) were analysed. **Results**: The inclusion criteria were met by 27,531 patients. The majority of seriously injured patients (*n* = 23,007) were transported to certified trauma centres without certification as ATZ. A total of 4524 patients were transported to a trauma centre with additional ATZ certifications. Mortality and the Revised Injury Severity Classification II (RISC-II) model for prediction of mortality after trauma were higher in ATZ hospitals. Logistic regression analysis showed no effect on mortality by a certification as a centre for geriatric trauma in treating seriously injured elderly patients. **Conclusions:** We assume that the additional ATZ certification does not positively influence the treatment of seriously injured elderly patients. A potential side effect could not be measured.

## 1. Introduction

The number of seriously injured elderly patients is rising significantly due to the demographic shifts toward an aging global population. The World Health Organization (WHO) predicts that by 2050, 1 in 6 people worldwide will be aged 65 years or older [1]. With increasing age comes a heightened vulnerability to trauma due to factors like pre-existing conditions and decreased physiological reserves. These factors complicate trauma management and contribute to higher mortality and morbidity rates in the elderly, even after less severe trauma [2]. In fact, the average age of trauma patients included in the TraumaRegister DGU^®^ (TR-DGU) exceeded age 54 in 2020, with nearly one-third (29.4%) aged 70 years or older. The overall mortality was reported as 7.4% in 2021 [3], but it is more than 20% for patients over 65 years [4]. This risk of death increases dramatically with age, particularly in polytraumatized patients. For instance, a Dutch study found that patients aged 70 years and older had a ninefold higher risk of dying compared to those aged 25–49 years [5]. Years before the first white paper on geriatric trauma was published, a single-centre study of a Level 1 trauma centre in Germany revealed a mortality rate of 57.4% in seriously injured patients aged 75 years and older, compared to an overall mortality rate of 28.7% [6,7]. To close this gap, the last update of the German S3 polytrauma guideline recommends a more liberal alert in emergency rooms with geriatric patients and addresses some specific physiological characteristics in this cohort. Seriously injured patients were to be transported to a suitable trauma centre. A prehospital differentiation in age regarding the hospital’s expertise in geriatric care has not been analysed thus far and is not part of the S3 polytrauma guidelines [8].

One of the most significant developments in geriatric trauma care has been the establishment of orthogeriatric co-management models. These multidisciplinary approaches, initially implemented for patients with proximal femur fractures, have demonstrated clear benefits in terms of reduced mortality and improved functional outcomes [9,10,11]. The basis of this orthogeriatric co-management is a certification as a Centre for Geriatric Trauma (ATZ). The participating centres are obliged to provide orthogeriatric care with standard operating procedures and provide their data in the Registry for Geriatric Trauma (ATR-DGU). Data of patients with a fracture of the proximal femur are entered into the register, and geriatric care is mandatory for these patients. Especially in cases of mortality, a 20% decrease could be achieved for those patients with a single trauma [10].

Despite the demonstrated benefits of orthogeriatric care for specific injuries like hip fractures, it remains unclear whether similar advantages apply to geriatric patients with multiple injuries. A study by Peterer et al. (2019) showed that the implementation of standard operating procedures for managing geriatric trauma patients, without orthogeriatric involvement, resulted in reduced mortality, suggesting that systematized care processes alone could have a positive effect on outcomes [12].

However, there is a lack of robust data on the role of geriatricians in the care of polytraumatized elderly patients, raising questions about whether orthogeriatric co-management models can be extended to this more complex cohort. The TraumaRegister DGU^®^, which serves as a comprehensive repository for trauma data, does not currently collect information regarding the involvement of geriatricians in treating severely injured elderly patients. Therefore, the potential benefits of involving geriatricians in the care of polytrauma patients remain largely speculative.

We hypothesised that if a certified trauma centre is also a certified centre for geriatric trauma, an effect on mortality in treating seriously injured elderly patients could be measured. To test this hypothesis, we conducted a retrospective analysis using data from the TraumaRegister DGU^®^ to assess whether certification as an ATZ is associated with reduced in-hospital mortality and improved clinical outcomes for patients aged 70 years and older with severe injuries. This study aims to fill the gap in current literature regarding the efficacy of geriatric trauma centres in managing the complexities of polytraumatized elderly patients and to provide insights into whether the benefits of orthogeriatric co-management can be extended beyond single-system injuries like hip fractures.

## 2. Materials and Methods

### 2.1. Data Sources

Data were used from the TR-DGU, and additional data about certification as an ATZ was provided from the Academy of Trauma Surgery (AUC–Akademie der Unfallchirurgie GmbH) that hosts the TraumaRegister DGU^®^ and the Registry for Geriatric Trauma (ATR-DGU). The TraumaRegister DGU^®^ of the German Trauma Society (Deutsche Gesellschaft für Unfallchirurgie, DGU) was founded in 1993. The aim of this multicentre database is a pseudonymised and standardised documentation of seriously injured patients.

Data were collected prospectively at four consecutive time phases from the site of the accident until discharge from the hospital: (A) pre-hospital phase, (B) emergency room and initial surgery, (C) intensive care unit, and (D) discharge. The inclusion criterion required admission to hospital via emergency room with subsequent ICU/ICM care or arriving at hospital with vital signs and dying prior to admission to ICU.

The infrastructure for documentation, data management, and data analysis was provided by AUC—Academy for Trauma Surgery (AUC—Akademie der Unfallchirurgie GmbH)—a company affiliated with the German Trauma Society. Scientific leadership was provided by the Committee on Emergency Medicine, Intensive Care, and Trauma Management (Sektion NIS) of the German Trauma Society. The participating hospitals submitted their pseudonymised data into a central database via a web-based application. Scientific data analysis was approved according to a peer review procedure in the guidelines established by TraumaRegister DGU^®^.

The participating hospitals were primarily located in Germany (90%), but an increasing number of hospitals from other countries contributed data as well (at present, these include Austria, Belgium, China, Finland, Luxembourg, Slovenia, Switzerland, The Netherlands, and the United Arab Emirates). Currently, more than 38,000 cases from nearly 700 hospitals are entered into the database annually. Participation in TraumaRegister DGU^®^ is voluntary. For hospitals associated with TraumaNetzwerk DGU^®^, however, the entry of at least a basic data set is obligatory for quality assurance [13].

### 2.2. Patients

Primarily, patients admitted from Germany, Austria, and Switzerland documented in the TR-DGU between 2016 and 2021 qualified for analysis. Patients had to be 70 years or older, require intensive care, and the worst injury had to have an abbreviated injury scale (AIS) severity of three or higher.

Certain exclusions were applied to maintain the consistency and reliability of the results. Patients who were transferred out of the hospital within 48 h, which resulted in no final outcome, were excluded. Additionally, patients transferred in from another hospital were not included, as this could lead to inconsistencies in the data. Finally, to avoid bias in mortality outcomes, patients who passed away within six days and had a documented will to limit therapy were also excluded from the study. The remaining patients were then divided into two distinct subgroups, depending on whether or not the hospital where they were treated had certification as a Centre for Geriatric Trauma (ATZ).

### 2.3. Outcomes

The primary outcome of this study was in-hospital mortality. Several secondary outcome measures were also examined to provide a comprehensive analysis of patient recovery and health status. These included the total number of days patients were intubated, the length of their stay in the intensive care unit (ICU), and the overall duration of their hospitalization. Additionally, the discharge target—whether patients were discharged home or to a rehabilitation facility—was analysed, along with their functional recovery, assessed using the Glasgow Outcome Scale (GOS). The Revised Injury Severity Classification, version II score (RISC II), which consists of 13 variables, was listed as a calculated predictor of mortality [14].

### 2.4. Statistical Analysis

All calculations were performed via Statistical Package for Social Sciences (SPSS 29.0; IBM Inc., Armonk, NY, USA). For descriptive analyses, categorical data were presented as numbers and percentages and continuous variables as median with interquartile range (IQR). A logistic regression analysis of mortality in seriously injured patients was used to identify the influencing factors.

### 2.5. Ethics

Written patient consent was obtained from the participating hospitals. The present study is consistent with the publication guidelines of the TraumaRegister DGU^®^ (TR-DGU) and is registered under the TR-DGU project ID 2022-010. This study was performed in accordance with the ethical standards of the 1964 Declaration of Helsinki and its later amendments. According to the guidelines of the responsible state medical association of North Rhine, ethical approval was not necessary for this retrospective anonymous analysis.

## 3. Results

**Figure 1:**
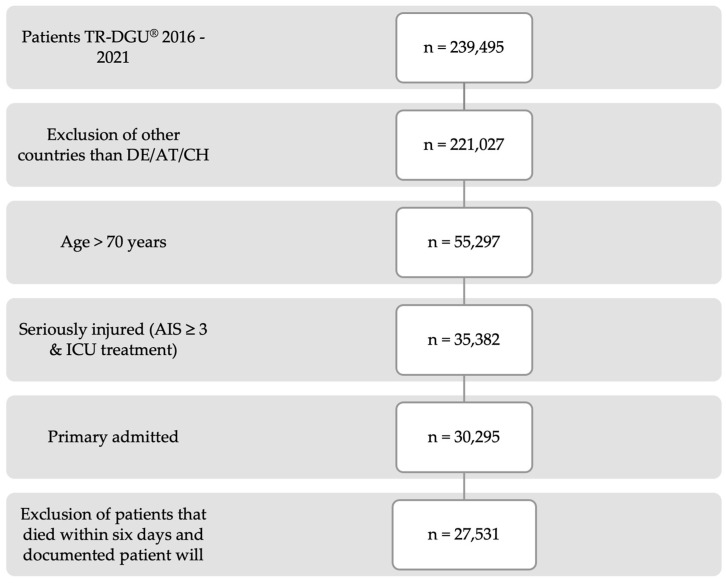
Flow sheet for patient inclusion (DE, Germany; AT, Austria; CH, Switzerland; AIS, abbreviated injury scale; ICU, intensive care unit).

After applying the inclusion criteria, a total of 27,531 patients from 700 certified hospitals within the TraumaNetzwerk DGU^®^ were selected for this study (Figure 1). Among these, 110 hospitals also held certification as Centres for Geriatric Trauma (ATZ). Of the 27,531 patients, 23,007 were transported to a certified trauma centre, while 4524 were transferred to a trauma centre with an additional ATZ certification. In total, the majority of patients were classified as ASA grade 2 (*n* = 10,709) and 3 (*n* = 11,390) preclinically; only 584 patients were classified as ASA grade 4 and 1.939 as ASA grade 1.

**Figure 2:**
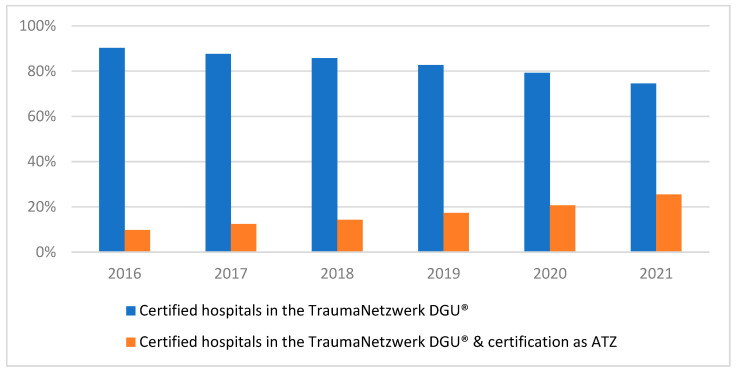
Development of the distribution of the included patients to the hospitals over time (years).

These patients were then divided into two groups based on whether the hospital had an ATZ certification during the observation period. Over the course of the study period, the proportion of patients treated at ATZ-certified centres steadily increased. In 2016, less than 10% of patients were treated at these centres, but by 2021, this figure had risen to 25.5% (Figure 2).

Despite this increase, the majority of seriously injured patients continued to be treated at trauma centres without ATZ certification. The distribution of patients in terms of age and sex remained relatively consistent between both groups, with only slight differences observed. Certified ATZ hospitals received more low fall trauma patients and fewer car accident patients, but the injury severity score (ISS) was nearly the same in both groups. The Revised Injury Severity Classification II (RISC II), as a predictor for mortality, was higher in the ATZ hospitals, and a higher mortality was observed. A higher proportion of surviving patients in ATZ-certified hospitals were discharged to a rehabilitation unit compared to those in non-ATZ certified hospitals. The mean hospital stay and invasive ventilation were the same in both groups (Table 1).

A second analysis was performed to evaluate the differences in patient outcomes at hospitals newly certified as Centres for Geriatric Trauma (ATZ) during the observation period between 2016 and 2021. In these newly certified ATZs, the analysis revealed that the patients were generally older, and there was a higher proportion of male patients. Regarding the types of trauma, more patients suffered from high-fall-related injuries, while there was a relative decrease in trauma cases involving car accidents. However, the overall distribution of other types of injuries remained similar across the patient population.

In the newly certified hospitals, a higher injury severity score (ISS) was observed, indicating more severe trauma among the patients. Additionally, the Revised Injury Severity Classification II (RISC II) score, which predicts mortality risk, was also higher in these newly certified ATZs. This corresponds to an increase in mortality rates, as more patients died during their hospital stay compared to hospitals that had not recently obtained ATZ certification. However, the Glasgow Outcome Scale and the discharge destination for surviving patients did not differ between the groups. The mean hospital stay, duration of invasive ventilation, and number of days in the intensive care unit were nearly the same in both groups. These findings are presented in Table 2, highlighting key differences in patient outcomes between those hospitals.

To determine the influence of a certification as a centre for geriatric trauma on mortality, a logistic regression analysis was performed. After applying the adjustments (RISC II, level of hospital’s care, transport, ASA grade, sex), no benefit from certification could be seen (OR: (95% CI) 1.076 (0.975–1.187), *p* = 0.146).

## 4. Discussion

The influence of orthogeriatric care in treating seriously injured elderly patients has not yet been analysed. Orthogeriatric care was initially designed to improve outcomes for elderly patients who require orthopaedic surgery, particularly those with fragility fractures such as proximal femur fractures [15]. From previous studies, we know that orthogeriatric co-management, which involves both orthopaedic surgeons and geriatricians working together, has a positive impact on survival rates for patients with hip fractures. This collaborative care model addresses not only the surgical needs of elderly patients but also their complex comorbidities and frailty, which can significantly influence recovery. [7,10,16,17]. In this analysis, we observed that the mandatory implementation of orthogeriatric care following the certification of hospitals as Centres for Geriatric Trauma (ATZ) did not result in a reduction in mortality rates among seriously injured elderly patients. The introduction of this certification, which aims to integrate specialized geriatric care into trauma management, did not appear to offer additional benefits in terms of survival outcomes when compared to non-certified trauma centres. This finding is significant, as it challenges the assumption that certification alone, without further refinement in care protocols, automatically improves clinical outcomes for elderly trauma patients.

Moreover, an interhospital comparison of centres that received ATZ certification during the observation period further supports this conclusion. Hospitals that were newly certified during the study period did not demonstrate higher survival rates or an increased frequency of patient discharge to rehabilitation facilities compared to non-certified hospitals. This suggests that while certification mandates the inclusion of geriatric care protocols, it does not necessarily translate to measurable improvements in key patient outcomes, such as mortality or functional recovery. It is important to acknowledge several factors that may have influenced these findings. First, the proportion of patients with higher predicted mortality, as indicated by the Revised Injury Severity Classification II (RISC II) score, was greater in ATZ-certified centres. This suggests that these centres may have treated a higher-risk patient population, which could partly explain why no significant mortality reduction was observed despite the certification. Additionally, we noted a higher proportion of male patients and cases involving high fall accidents in newly certified centres. Male gender, for example, has been associated with increased mortality in trauma [18,19].

In addition, the mortality rates observed in this study were notably higher than what the RISC II model had predicted. Several factors may explain this discrepancy, particularly in the context of elderly trauma patients. It is known that advanced age, combined with a higher injury severity score (ISS), is associated with a greater likelihood of palliative care consultation and decisions to withdraw aggressive treatment. These factors can lead to increased mortality rates, as care may shift toward comfort measures rather than curative interventions in severely injured older patients [20,21]. This had not been considered when the RISC II model was developed. As a result, the model may underestimate the mortality risk in elderly patients who are more likely to opt for, or be recommended, palliative care.

Another limitation of the RISC II model is the way it handles age as a predictor. Due to the limited sample size available during the model’s development, patients aged 85 years and older were grouped into a single risk category. This simplification likely leads to an underestimation of mortality risk in the oldest patients. Recent studies have indicated that mortality increases significantly starting from age 55 and older, so that the mortality rate in the very old patients might be underestimated [14].

We assume that the patients included in this study were admitted to hospitals based primarily on the pattern and severity of their injuries, as prehospital triage or selection based solely on age is not currently part of any known guidelines for trauma management. Instead, triage decisions are generally guided by the nature of the injury and its severity. Most of the patients in this analysis were transported to a Level 1 trauma centre, which provides comprehensive care with a multi-professional medical team available around the clock. The literature consistently supports the benefit of treating seriously injured patients in Level 1 trauma centres, where the availability of specialized staff, advanced technology, and multidisciplinary care improve patient out-comes [22].

Relevant differences in the baseline data between both groups were not found. In contrast to most other studies regarding trauma of the elderly, male patients constitute the majority of seriously injured elderly patients [23].

Additionally, the type of accidents leading to hospital admissions in the elderly population differed substantially from the overall trauma data. More than half of the elderly patients in this study were admitted due to low-fall accidents, which stands in stark contrast to the general data from the TraumaRegister DGU^®^, where only 25% of trauma admissions are attributed to falls [3]. This highlights the nature of injury patterns in the elderly population, where even low-energy traumas, such as falls from a standing height, can lead to serious injuries due to factors such as frailty, osteoporosis, and other age-related physiological changes.

Although low-fall accidents are the main cause for seriously injured patients and for fragility fractures of the hip, the influence of the geriatrician on mortality differs. The genesis of the injuries and the concomitant suggestibility of the disease progress through a geriatrician should be discussed. We assume that most patients with a hip fracture suffer from typical geriatric comorbidities like chronic kidney disease, diabetes mellitus, osteoporosis, glucocorticoid treatment, and low body mass index which all increase the risk for fragility fractures of the hip [24,25,26]. Given this, the involvement of a geriatrician could potentially play a critical role in managing these comorbidities. Geriatricians bring specialized knowledge of the physiological changes associated with aging, which may help mitigate some of the risks linked to chronic conditions and improve overall patient management. For example, interventions to address osteoporosis, better control of diabetes, and optimizing kidney function could contribute to better outcomes in trauma patients with pre-existing conditions. However, in this analysis, we were unable to measure any effect of an additional certification as a Centre for Geriatric Trauma on reducing mortality in elderly patients with serious trauma. The lack of measurable impact could be due to various factors, including the heterogeneity of the patient population or the complexity of polytrauma cases in which multiple organ systems are affect.

Despite the potential benefits of geriatric co-management in orthopaedic cases, such as those involving hip fractures, the role of geriatricians in the acute management of trauma patients has not been well studied. Future research should aim to assess the influence of geriatricians on trauma care outcomes more comprehensively, especially in cases of acute trauma, where their expertise could address the unique needs of elderly patients beyond standard orthopaedic care.

## 5. Conclusions

It can be concluded that the mandatory implementation of orthogeriatric care through Centres for Geriatric Trauma (ATZ) certification did not lead to a significant reduction in mortality rates among seriously injured elderly patients. Although the integration of specialized geriatric care in trauma management has been shown to improve outcomes in certain orthopaedic contexts, such as hip fractures, the certification alone did not demonstrate additional benefits in survival rates or functional recovery when compared to non-certified trauma centres. Moreover, the observed higher mortality rates than predicted by the RISC II model indicate the complex interplay of factors influencing outcomes in elderly trauma patients, including the prevalence of comorbidities, the role of palliative care, and the challenges in risk prediction models for this population. This study underscores the need for further research to better understand the role of geriatricians in the acute care of trauma patients and to explore the potential benefits of more targeted interventions to improve survival and recovery in this vulnerable group.

## 6. Limitations

This study has several limitations that should be acknowledged. First, it was a retrospective study, which inherently carries the risk of known potential biases, such as selection bias and confounding. Although the data from the TraumaRegister DGU (TR-DGU) were collected prospectively, the retrospective analysis introduces the possibility that not all variables were captured or accurately reflected in the dataset. This is a common issue with retrospective studies, where the reliance on existing data may limit the ability to control for unmeasured confounding factors. Despite these challenges, the data collection process for the TR-DGU is robust, and quality protection is maintained through certifications and audits at all participating hospitals. However, the quality and completeness of data depend on accurate entry by hospital staff, which can vary across centres.

Another significant limitation is the lack of specific data regarding whether a seriously injured patient was seen by a geriatrician. The TR-DGU does not include a query to track whether patients received orthogeriatric co-management, making it difficult to assess the true impact of geriatrician involvement in trauma care. We assume that once a hospital is certified as a Centre for Geriatric Trauma (ATZ), it follows the prescribed orthogeriatric care protocols, but we cannot verify the consistency or extent of orthogeriatric co-management implementation across different hospitals. This lack of detailed information on how geriatric care is integrated may introduce a bias, as the actual quality and level of geriatric care could vary significantly between centres.

Furthermore, the exclusion of patients who died within six days and had a documented patient will to limit therapy is another limitation. These patients may have had a different prognosis or injury severity compared to the included population, and their exclusion could bias the results. Since we do not know whether these patients belonged to the group treated at ATZ-certified hospitals or non-certified hospitals, their exclusion may affect the generalizability of the findings. This is particularly relevant in geriatric trauma populations, where end-of-life care decisions are more common due to the higher prevalence of comorbidities and frailty, which may influence both the decision to pursue aggressive treatment and overall survival outcomes.

Lastly, this study’s reliance on the RISC II model for mortality prediction, which has its limitations in elderly populations, introduces a further constraint. The RISC II model was not specifically designed to account for the physiological complexity of elderly patients or factors like palliative care decisions, which could skew mortality outcomes. Future studies could benefit from using more refined models that take these variables into consideration.

## Figures and Tables

**Table 1:** Comparison of certified hospitals in the TraumaNetzwerk DGU^®^ with or without additional certification of ATZ; ISS, injury severity score; ICU, intensive care unit; d, days; SD, standard deviation; RISC II, Revised Injury Severity Classification II.

	No ATZ	ATZ
Number of patients	23,007	4524
Hospital‘s level of care		
1	12,838 (55.8%)	2914 (64.4%)
2	7800 (33.9%)	1431 (31.6%)
3	2369 (10.3%)	179 (4.0%)
Age		
Mean (SD)	80 (±6.2)	80.3 (±6.3)
Median	79	80
Sex		
Female	9727 (42.3%)	1894 (41.9%)
Male	13,280 (57.7%)	2630 (58.1%)
Accident		
Car	3005 (13.2%)	455 (10.3%)
Motor bike	600 (2.6%)	117 (2.6%)
Bicycle	2246 (9.9%)	406 (9.2%)
Pedestrian	1428 (6.3%)	246 (5.5%)
High fall	2719 (12%)	545 (12.3%)
Low fall	11,580 (51%)	2435 (54.9%)
other	1141 (5%)	233 (5.3%)
ISS		
Mean (SD)	20.5 (±10.2)	20.4 (±9.9)
Median	18	18
Hospital stay (d)		
Mean (SD)	16.4 (±16.3)	17.1 (±17.1)
Median	13	13
ICU treatment (d)		
Mean (SD)	7.4 (±10.2)	7.8 (±10.5)
Median	3	3
Intubation (d)		
Mean (SD)	3.2 (±7.8)	3.4 (±7.5)
Median	0	0
Discharge destination (survivor only)		
Home	8881 (48.8%)	1668 (47.8%)
Rehabilitation unit	5455 (30.0%)	1156 (33.1%)
Other hospital	2427 (13.3%)	405 (11.6%)
Other	1449 (8.0%)	262 (7.5%)
Glasgow Outcome Scale (GOS) (survivor only)		
Persistent vegetative state	372 (2.1%)	91 (2.6%)
Severe disability	2383 (13.2%)	469 (13.6%)
Moderate disability	5834 (32.4%)	1131 (32.7%)
Good recovery	9409 (52.3%)	1765 (51.1%)
Hospital mortality	4795 (20.8%)	1033 (22.8%)
95% confidence interval	20.3–21.4%	21.5–24.3%
Expected mortality		
(based on RISC II)	20.8%	21.5%

**Table 2:** Comparisons of hospitals in the TraumaNetzwerk DGU^®^ that became certified as ATZs between 2016 and 2021. ISS, injury severity score; ICU, intensive care unit; d, days; SD, standard deviation; RISC II, Revised Injury Severity Classification II.

	Before Certification as ATZ	After Certification as ATZ
Number of patients	2078	2180
Hospital‘s level of care		
1	1374 (66.1%)	1429 (65.6%)
2	557 (26.8%)	643 (29.5%)
3	147 (7.1%)	108 (5.0%)
Age		
Mean (SD)	79.9 (±6.2)	80.2 (±6.1)
Median	79	80
Sex		
Female	872 (42%)	854 (39.2%)
Male	1206 (58%)	1326 (60.8%)
Accident		
Car	277 (13.4%)	202 (9.4%)
Motor bike	62 (3%)	67 (3.1%)
Bicycle	173 (8.4%)	193 (9%)
Pedestrian	124 (6%)	104 (4.9%)
High fall	227 (11%)	300 (14%)
Low fall	1094 (53.1%)	1142 (53.3%)
other	105 (5.1%)	133 (6.2%)
ISS		
Mean (SD)	20.4 (±10)	21 (±10.3)
Median	17	18
Hospital stay (d)		
Mean (SD)	16.8 (±16.3)	16 (±15.1)
Median	13	12
ICU treatment (d)		
Mean (SD)	8.1 (±11.9)	7.7 (± 10.3)
Median	3	4
Intubation (d)		
Mean (SD)	3.7 (±8.9)	3.4 (±7.2)
Median	0	0
Discharge destination (survivor only)		
Home	752 (46.1%)	754 (46.2%)
Rehabilitation unit	567 (34.8%)	573 (35.1%)
Other hospital	186 (11.4%)	188 (11.5%)
Other	125 (7.7%)	118 (7.2%)
Glasgow Outcome Scale (GOS) (survivor only)		
Persistent vegetative state	35 (2.2%)	40 (2.5%)
Severe disability	242 (15.0%)	247 (15.4%)
Moderate disability	523 (32.3%)	526 (32.7%)
Good recovery	817 (50.5%)	796 (49.5%)
Hospital mortality	448 (21.6%)	547 (25.1%)
95% confidence interval	19.6–23.7%	23.0–27.3%
Expected mortality (based on RISC II)	21.7%	23.7%

## Data Availability

The original contributions presented in the study are included in the article, further inquiries can be directed to the corresponding author.
